# Myelitis: A Common Complication of Tuberculous Meningitis Predicting Poor Outcome

**DOI:** 10.3389/fneur.2022.830029

**Published:** 2022-03-16

**Authors:** Yuxuan Jiang, Xiangqin Xu, Zhuoxin Guo, Yuxin Liu, Jiahao Lin, Lijun Suo, Ying Jiang, Bo Liu, Tingting Lu

**Affiliations:** ^1^Department of Neurology, The Third Affiliated Hospital of Sun Yat-sen University, Guangzhou, China; ^2^Department of Radiology, The Third Affiliated Hospital of Sun Yat-sen University, Guangzhou, China; ^3^Zibo City Key Laboratory of Respiratory Infection and Clinical Microbiology, Department of Pulmonary and Critical Care Medicine, Zibo City Engineering Technology Research Center of Etiology Molecular Diagnosis, Zibo Municipal Hospital, Zibo, China

**Keywords:** tuberculous meningitis, myelitis, paradoxical reaction, immune reaction, prognosis

## Abstract

**Background:**

Myelitis is an important complication in patients with tuberculous meningitis (TBM). However, a paucity of publications exists on the spectrum of neurological and MRI findings of TBM-related myelitis. The risk factors and prognosis of myelitis in patients with TBM are not fully understood. Therefore, this study aims to identify the risk factors, clinicoradiological features, and prognostic impact of myelitis for patients with TBM.

**Methods:**

We conducted a retrospective study in our institution. Patients with TBM who were consecutively admitted during the period of August 2015 to December 2019 were included. We reviewed the demographic characteristics, clinical, laboratory and MRI findings, and clinical outcomes of all of the included patients. The diagnosis of myelitis was identified by a hyperintensity on T2-weighted images that were associated with cord edema, enlargement, and marginal or no enhancement on contrast-enhanced images.

**Results:**

A total of 114 patients were included. Myelitis occurred in 19 (16.7%) patients, five of whom paradoxically developed myelitis. The common clinical signs of myelitis were paraparesis (738.9%), quadriparesis (844.4%), urinary retention or constipation (1,477.8%), and paresthesias in the lower limbs (1,052.6%). In the MRI findings, the hyperintensities on T2-weighted images involved more than 3 spinal cord segments. Myelitis was often combined with other forms of spinal cord injury, including 10 patients (52.6%) with spinal meningeal enhancement, 7 patients (36.8%) with enlargement of the central canal of the spinal cord, 6 patients (31.6%) with tuberculoma, and 4 patients (21.1%) with arachnoiditis and 1 patient (5.3%) with cerebrospinal fluid (CSF) loculations. None of the 5 patients with paradoxical myelitis were complicated with spinal meningeal enhancement and arachnoiditis, while 4 patients were complicated with enlargements of the central canal of the spinal cord. In multivariable analysis, a grade III disease severity on admission [*p* = 0.003, odds ratio (OR) = 8.131, 95% CI: 2.080–31.779] and high CSF protein (*p* = 0.033, OR = 1.698, 95% CI: 1.043–2.763) were independent risk factors for myelitis. After the 6 months follow-up, myelitis (*p* = 0.030, OR = 13.297, 95% CI: 1.283–137.812) and disturbance of consciousness (*p* = 0.042, OR = 12.625, 95% CI: 1.092–145.903) were independent risk factors for poor outcomes.

**Conclusion:**

Myelitis was a common complication of TBM and independently predicted a poor outcome. A grade III disease severity and high CSF protein on admission were independent risk factors for myelitis. Paradoxical myelitis was rarely complicated with spinal meningeal enhancements and arachnoiditis, indicating that the immune reaction may play a dominant role.

## Introduction

Among infectious diseases, tuberculosis (TB) is the leading cause of death worldwide. The WHO declared that globally in 2019, 10.0 million (range, 8.9–11.0 million) people fall ill with TB (1). Tuberculous meningitis (TBM) is a kind of extrapulmonary TB that accounts for 1% of all the tuberculous cases and is characterized by subacute or chronic meningitis due to the invasion of *Mycobacterium tuberculosis* (MTB) into the subarachnoid space. Even after standard treatment, the prognosis of TBM is still poor. Chronic neurological dysfunction is common (2). Approximately 10% of patients with TBM develop spinal TB. Vertebral body TB (Pott's disease) with cord impingement accounts for the majority of cases. Spinal cord involvement in TBM is rare and less reported (3).

The earliest evidence of spinal cord involvement in TBM came from an autopsy. Cameron provided an early description of myelopathy with spinal subarachnoid obstruction secondary to TBM in 1919 (4). With the advances in neuroimaging, researchers have found a variety of spinal cord-related complications, including tuberculous radiculomyelitis, spinal tuberculoma, myelitis, syringomyelia, and spinal tuberculous abscesses (5–8).

Myelitis is one of the less noticed complications of TBM. It has been defined as a hyperintense signal on T2-weighted images with spinal cord edema and enlargement and a marginal enhancement on postcontrast MR images (9). In the past, TBM with myelitis has been mostly reported in the form of case reports (6, 10–14). In these case reports, a total of 15 patients had been diagnosed with TBM-related myelitis. Fourteen (93.3%) of them were initially hospitalized with symptoms of myelitis. Interestingly, 1 (6.7%) patient developed new symptoms of myelitis during anti-TB treatment. After standard anti-TB treatment, 9 (60%) patients recovered completely, 3 (20%) were left with slight neurological defects, and another 3 (20%) were left with severe neurological defects. In a prospective study, 33/71 TBM patients were complicated with spinal cord and nerve root involvement. Myelitis was diagnosed in 14 patients. A systematic review including 147 patients with TBM with spinal cord-related complications found that 8.84% of patients had acute transverse myelitis and longitudinally extensive transverse myelitis (15). However, the manifestations, related factors, and prognostic significance of myelitis were not mentioned (16). Given the unique pathophysiological mechanism of myelitis, especially since it is possibly associated with an abnormal immune response to infection, it is worthwhile to study myelitis separately. The onset forms of myelitis could either be initial or paradoxical. The distinct characteristics and risk factors of these forms of myelitis also need to be clarified.

In this study, we retrospectively evaluated the incidence, MR characteristics, predictors, and prognosis of patients with TBM with myelitis.

## Methods

### Patient Population

We retrospectively reviewed the clinical, laboratory, and imaging data of patients with TBM consecutively admitted to the Third Affiliated Hospital of Sun Yat-sen University from August 2014 to December 2019. This study was approved by the ethics committee of the Third Affiliated Hospital of Sun Yat-sen University.

### Diagnostic Criteria for TBM

Tuberculous meningitis was diagnosed according to the consensus diagnostic criteria published in 2010 (17). According to their total diagnostic score, patients were diagnosed with definite, probable, or possible cases. Definite cases were diagnosed based on acid-fast bacilli seen directly in the cerebrospinal fluid (CSF) and MTB was cultured or detected by a reliable molecular method from the CSF. Probable TBMs were cases with a diagnostic score ≥ 12 (at least 2 points either came from the CSF or the cerebral imaging criteria) or a diagnostic score of ≥10 when imaging was not available. Patients were diagnosed with possible TBM if their diagnostic score was 6–11 or 6–9 when imaging was not available.

### Inclusion and Exclusion Criteria

Inclusion criteria: All the consecutive newly diagnosed patients with TBM fulfilling the consensus diagnostic criteria as described by Marais et al. were included. The exclusion criteria were as follows: (1) patients with clinical evidence of spinal cord involvement who had not undergone spinal cord MRI and (2) patients with a history of spinal cord injury, myelitis, and other myelopathy.

### Demographic and Clinical Assessment

The demographic features that were collected included age and sex. The clinical features included the patient's symptoms, such as hemiparesis, paraparesis, paresthesias/pain in lower limbs, quadriparesis, urinary symptoms, constipation, altered consciousness, cranial nerve palsy, and seizure; history of TB/abnormal chest X-ray; and related concomitant diseases such as diabetes, hepatitis B virus (HBV) infection, autoimmune diseases and metabolic syndrome. The severity of TBM was graded as grade I, grade II, or grade III. Patients who were fully conscious or had non-specific symptoms were graded as grade I. Patients with lethargy or cranial nerve palsy symptoms were graded as grade II. Patients with severe illness, gross paralysis, or paresis were graded as grade III (18). Disturbance of consciousness was defined as the Glasgow Coma Scale (GCS) score of ≤8 (19). We asked each patient whether they had a weakness, pain, paresthesias in the lower limbs, urinary retention or incontinence, and constipation. We carried out detailed neurological examinations, including the determination of reflex changes, tone changes, discrete power on the Medical Research Council scale, extender plant response, and sensor loss. Spinal cord MR examinations were recommended for patients with hemiparesis, paraparesis, paresthesia/pain in the lower limbs, quadriparesis, urinary symptoms, and constipation.

### Cerebral and Spinal Imaging

Brain MRI scans were performed in all the patients. Eligible patients underwent spinal cord MR examination for routine clinical diagnostic purposes using a 3.0 T/1.5 T MR scanner (Discovery MR750/360, GE). For brain imaging, T1, T2, T1 contrast images, diffusion-weighted imaging (DWI), and magnetic resonance angiography (MRA) were obtained. For spinal imaging, T1 and T1 contrast and T2 were obtained. An experienced neuroradiologist and a neurologist, both of whom were blinded to the patients' diagnosis and clinical features, analyzed all of the MRI scans. The final assessments were made by consensus. The presence of intracranial infarcts, hydrocephalus, basal exudates, tuberculomas, myelitis, arachnoiditis, tuberculoma, and atrophy in the spinal cord was documented. The diagnosis of myelitis was identified by hyperintensities on T2-weighted images associated with cord edema, enlargement, and marginal or no enhancement on contrast-enhanced images (9).

### Treatment

The initial anti-TB treatment regimen for all patients was oral isoniazid (600 mg/day), rifampicin (450 mg/day), pyrazinamide (1.5 g/day), and ethambutol (15 mg/kg). During treatment, the patient's treatment was adjusted following the recommendations of the guidelines according to drug-related adverse reactions, TB resistance, and other factors. Although the change in treatment regime could affect the prognosis of patients, all the patients accepted the optional optimal treatment. The total course of anti-TB treatment was at least 12 months (3). Most patients received intravenous dexamethasone therapy for 4 weeks (0.4 mg/kg/day for week 1, 0.3 mg/kg/day for week 2, 0.2 mg/kg/day for week 3, and 0.1 mg/kg/day for week 4) and then oral treatment for 4 weeks starting at 4 mg/day, tapering by 1 mg each week. In patients with steroid intolerance or contraindications such as peptic ulcers, severe hypertension, and uncontrolled diabetes, steroids were administered using a smaller dose with a short course. In patients with severe symptoms or paradoxical reactions, high-dose steroid pulse therapy was used or the treatment duration was extended (20).

### Clinical Outcome Assessment

The specific form of follow-up depended on the patient's condition. Patients with severe conditions needed to be hospitalized for reexamination, while the other patients had regular outpatient or telephone follow-up. A paradoxical reaction was defined as the deterioration of the original TB lesions, the appearance of new lesions on MRI, or a transient worsening in the CSF parameters in patients whose clinical symptoms were initially improved with anti-TB treatment. The presentation of paradoxical reactions on MRI included the expansion of the existing brain and spinal cord lesions, the appearance of new tuberculomas, optochiasmatic arachnoiditis, hydrocephalus, and spinal cord injury (21). Paradoxical myelitis was defined as the presence of new symptoms of spinal cord injury during anti-TB treatment, and spinal cord MRI was confirmed as myelitis. The outcomes were assessed using the modified Barthel index (mBI) 6 months after the start of anti-TB treatments. Previous studies on TBM determined that an mBI ≥ 12 with a total score of 20 was associated with a good outcome. Considering that it is more common to use the mBI scale with a total score of 100, we generated the receiver operating characteristic curve (ROC). Then, we used a threshold of 60 with the biggest Youden index. Therefore, the patients were classified as poor outcomes (mBI <60 or death) and good outcomes (mBI score ≥ 60) (22–24).

### Statistical Analysis

We used Statistical Package for the Social Sciences 19 version software (IBM Corporation, Armonk, New York, USA) to perform statistical analysis. Continuous variables accepted the Shapiro–Wilk test, which conforms to the normal distribution or approximate normal distribution were expressed as mean ± SD and the Student's *t*-test was used to compare the mean difference between the two groups. The non-normal distribution variables were expressed by median (interquartile interval) and the median differences between the two groups were compared by Mann–Whitney *U* test. Categorical variables were expressed in counts (percentages) and the Pearson's chi-squared test was used to compare the difference between the two groups. We used univariate logistic regression analyses to screen variables and used the stepwise backward method of multivariate logistic regression analyses to determine the predictors for variables with *p* < 0.05 in the univariate analysis. A two-tailed *p* < 0.05 was considered statistically significant.

## Results

### Participants

**Figure 1:**
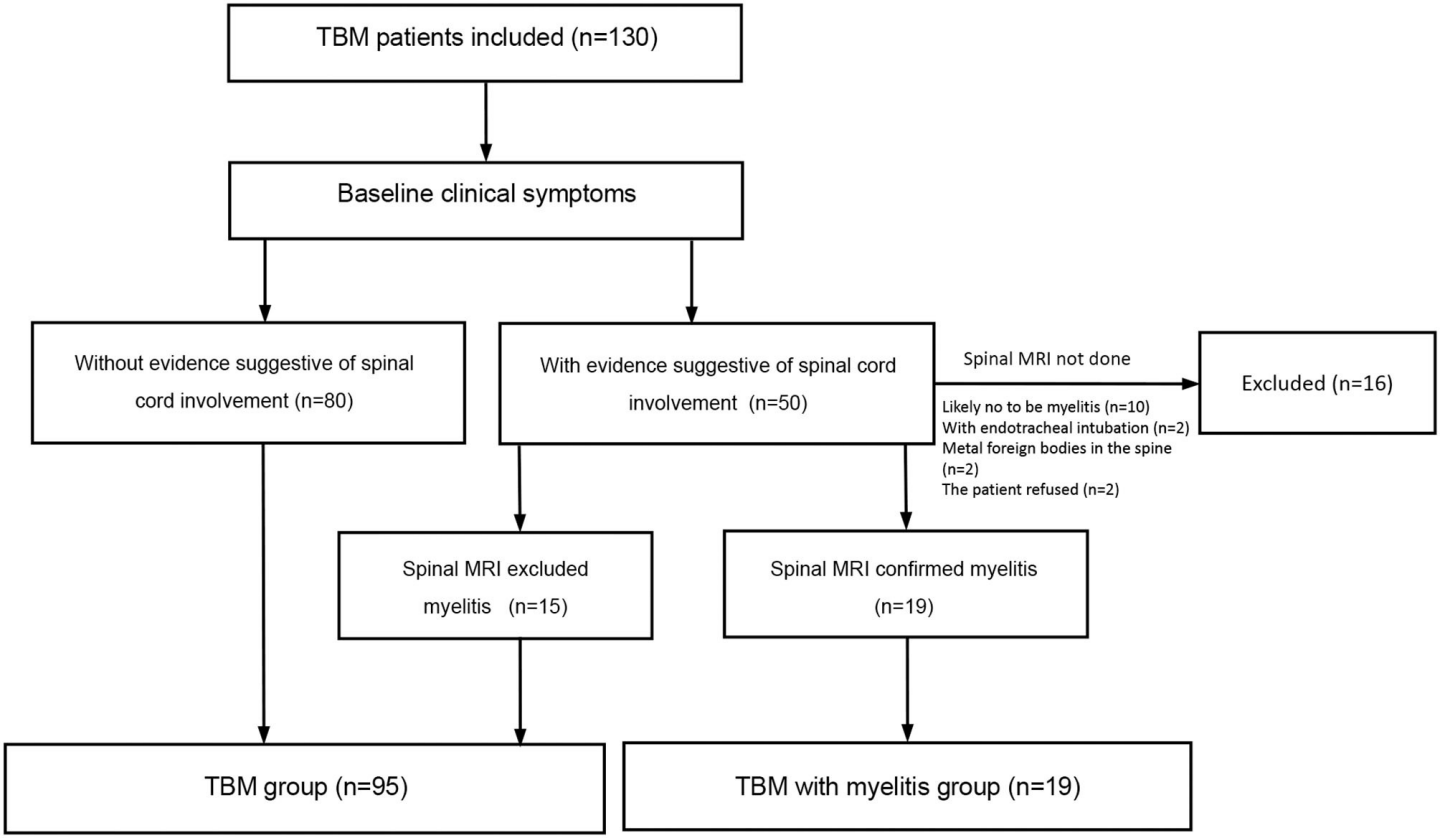
Flow chart of the study. The common signs suggesting spinal cord or spinal nerve root involvement include weakness, pain and paresthesia in the lower limbs or all the four limbs, urinary retention, and constipation.

As shown in Figure 1, a total of 130 patients with TBM were potentially included. Fifty patients had spinal cord involvement and symptom evaluations and 34 patients underwent spinal cord MR examinations. We excluded 16 patients who did not undergo spinal cord MR because of the following: (1) They were not likely to have myelitis (*n* = 10); (2) They had endotracheal intubation (*n* = 2); (3) Metal foreign bodies were found in the spine; (4) The patient refused an MRI for economic reasons. Finally, 114 patients with TBM were included. Myelitis was identified in 19 (16.7%) patients. The baseline demographic, clinical and radiological features, diagnostic category, and staging of TBM are described in Table 1.

**Table 1:** Baseline epidemiological, clinical, and neuroimaging characteristics of patients with tuberculous meningitis (TBM) (*n* = 114).

**Baseline findings**	**TBM without myelitis (Group1) (*n* = 95)**	**TBM with myelitis (Group2) (*n* = 19)**	**TBM with spinal (Group3) MR(*n* = 34)**	***P-values* (Group1 vs. Group2)**
Age ≥60	11 (12%)	1 (5%)	5 (15%)	0.682
**Sex**				
Male	64 (68%)	11 (58%)	20 (59%)	0.427
Time from onset to treatment start, median (range), day	14 (3–60)	14 (7–185)	15.5 (3–185)	0.625
Time from onset to first spinal MRI examnation, median (range), day	/	36 (7–195)	34 (7–195)	/
Extracranial tuberculosis	29 (31%)	3 (16%)	9 (26%)	0.192
Paradoxical reaction	41 (43%)	15 (79%)	25 (74%)	**0.004**
Disease severity				**<0.001**
Grade I	53 (56%)	5 (26%)	10 (29%)	
Grade II	30 (31%)	1 (5%)	9 (26%)	
Grade III	12 (13%)	13 (69%)	15 (45%)	
Disturbance of consciousness	3 (3%)	5 (26%)	5 (15%)	**0.002**
Cranial nerve damage	24 (25%)	5 (26%)	10 (29%)	1.000
Seizures	10 (11%)	4 (21%)	4 (12%)	0.372
**Initial brain MRI findings**				
Infarction	24 (25%)	6 (32%)	12 (35%)	0.605
Arterial inflammation	14 (15%)	6 (32%)	8 (24%)	0.166
Inflammatory nodules	13 (14%)	3 (16%)	3 (9%)	1.000
Hydrocephalus	12 (13%)	3 (18%)	5 (15%)	0.736
Basal exudates	43 (45%)	9 (47%)	15 (44%)	0.897
**Initial spinal MRI findings**				
Arachnoiditis	/	4 (21%)	6 (18%)	/
Spinal meningeal enhancement	/	10 (53%)	14 (41%)	/
Tuberculoma	/	6 (32%)	8 (24%)	/
Enlargement of central canal of spial cord	/	7 (37%)	8 (24%)	/
Cord atrophy	/	2 (11%)	2 (6%)	/
Cord swelling	/	3 (16%)	3 (9%)	/
Syrinx	/	0 (0%)	0 (0%)	/
CSF loculation	/	1 (5%)	1 (3%)	/
Initial CSF findings	/	4 (21%)	6 (18%)	/
Pressure/mmH2O (mean ± SD)	209.86 ± 86.73	243.61 ± 61.03	243.61 ± 45.03	0.121
Cell count, /ul (media IQR)	158 (241)	268 (308)	176 (280)	0.309
Protein, mg/dL (media IQR)	0.97 (0.95)	2.16 (2.43)	1.83 (2.31)	**0.005**
Sugar, mmol/L (mean ± SD)	2.42 ± 0.92	2.44 ± 1.44	2.41 ± 1.21	0.939
CL mmol/L (mean ± SD)	107.37 ± 6.34	115.82 ± 8.11	96.81 ± 7.22	0.558

*1Bold values indicate statistical significance (p < 0.05). Categorical varibles wERE analyzed using χ2 test or Fisher's exact test and quantitative data are evaluated using Mann–Whitney U-test or t-test with the least significant difference*.

*TBM, tuberculous meningitis; MRI, Magnetic Resonance Imaging; CSF, cerebrospinal fluid; Cl, chloride; IQR, interquartile range; SD, Standard Deviation*.

### Clinical Features of TBM With Myelitis

Among 114 patients, myelitis was diagnosed in 19 patients according to spinal MRI. The initial MRI indicated myelitis in fourteen (73.7%) patients, while five (26.3%) patients paradoxically developed myelitis during the treatments. The symptoms included weakness, pain, and paresthesia in the lower limbs or in all four limbs, urinary retention, and constipation. The common clinical signs on neurological examination were reflex changes, a decreased power on the Medical Research Council scale, an extensor plantar response, and sensory loss. Paraparesis was present in 7 (38.9%) patients. It was of the upper motor neuron (UMN) type in 5 patients (26.3%) and of the lower motor neuron type in 2 (10.5%) patients. Quadriparesis was present in 8 (44.4%) patients. It was of the UMN type in 5 (26.3%) patients, lower motor neuron type in 2 (10.5%) patients, and mixed type in 1 (5.3%) patient. Paresthesias in the lower limbs were present in 10 (52.6%) patients. Urinary retention or constipation was present in 14 (77.8%) patients. In 5 patients with paradoxical myelitis, the median time of myelitis was 1 week after treatment. Three patients had disturbances of consciousness at admission. The main clinical manifestations of myelitis were quadriparesis (2, 40%), paraparesis (3, 60%), and urinary retention and constipation (4, 80%).

### Magnetic Resonance Imaging Findings of Myelitis

**Figure 2:**
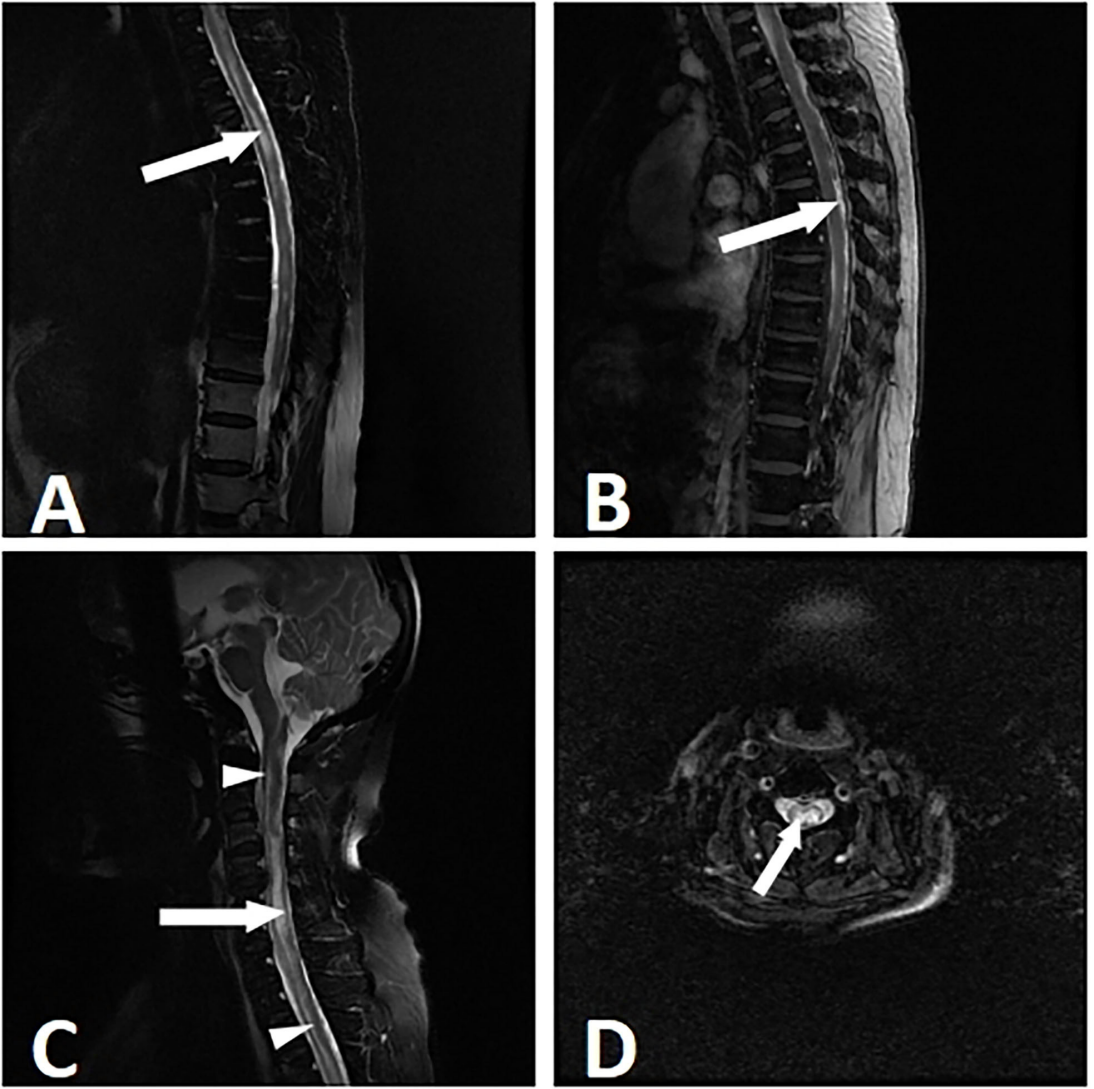
Myelitis in a 53-year-old woman with tuberculous meningitis. The patient suffered from paraparesis with the duration of 8 weeks. MRI showed longitudinally myelitis. T2-weighted imaging showed a stripped-like hyperintensity **(A)**. In the T1 contrast-enhanced sequence, uneven enhancements of the lesions were noticed **(B)**. T2-weighted sagittal and axial images showed an uneven enlargement of the spinal cord and syringomyelia. The syringomyelia manifested as a stripe of intramedullary near-water signals (long arrow) and enlargement of the central canal (short arrow) **(C,D)**.

**Figure 3:**
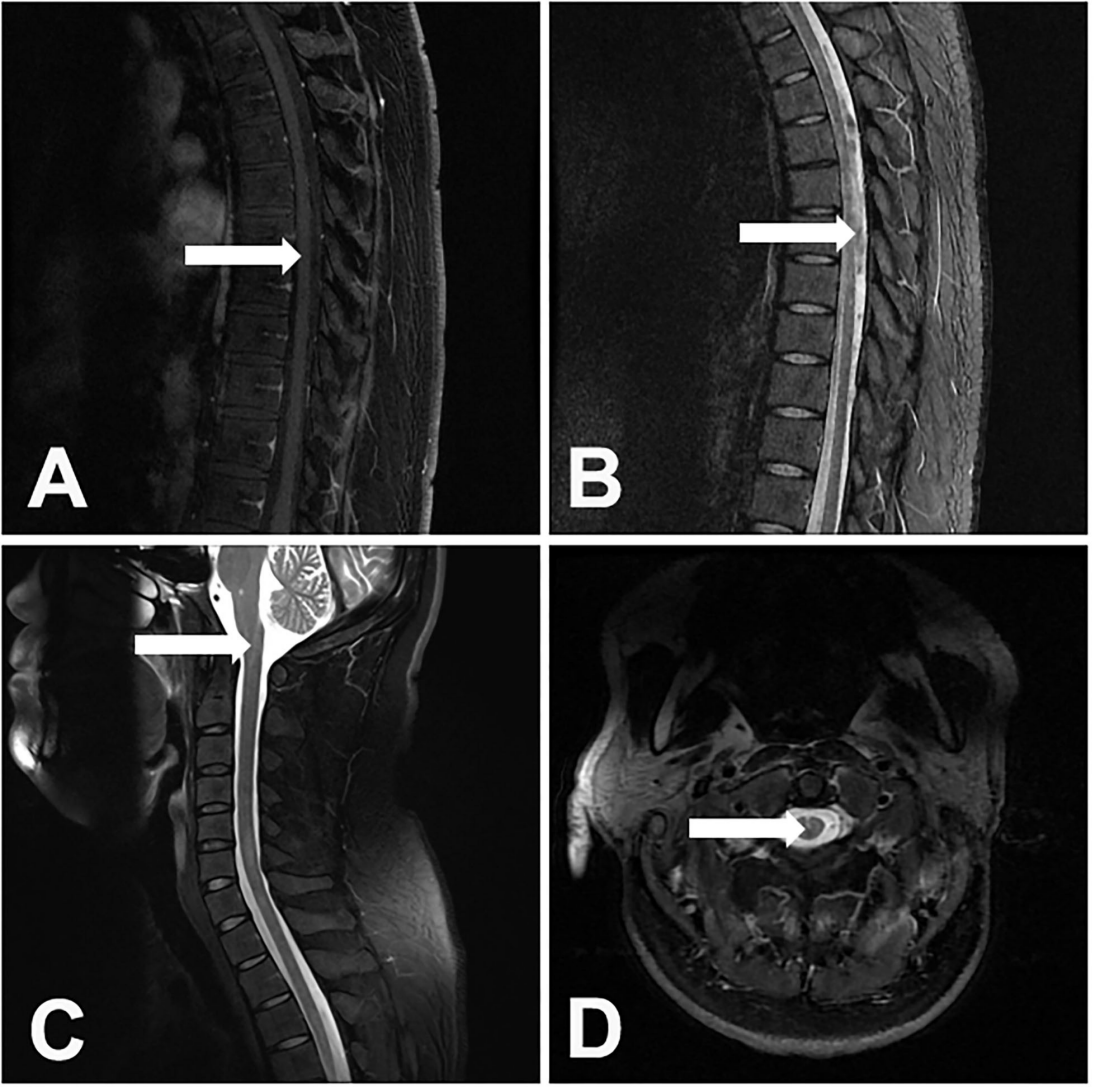
A 31-year-old male patient developed paradoxical myelitis. The patient developed new spinal cord lesions 3 months after the start of antituberculosis treatment, when his symptoms of headache, fever, and disturbance of consciousness were gradually alleviated. A T1-weighted sagittal image of the thoracic spinal cord of the patient showed a spot-like hypointensity **(A)**. In the T2-weighted sequence, multiple patchy hyperintensities in the spinal cord can be seen **(B)**. T2-weighted sagittal and axial images of the cervical spinal cord showed an enlargement of the central canal in the spinal cord **(C,D)**.

The cervical and thoracic spinal cord (10, 52.6%) were simultaneously involved, followed by the cervical (4, 21.1%), thoracic (3, 15.8%), and cervicothoracolumbar (2, 10.5%) regions. The lesions were present in more than 3 spinal cord segments in all of the patients. It should be noted that myelitis alone was rare. Only 1 (5.3%) patient developed isolated myelitis. The other patients were complicated with other spinal cord injuries, including 4 (21.1%) patients with arachnoiditis, 10 (52.6%) patients with spinal meningeal enhancement, 6 (31.6%) patients with tuberculomas, 7 (36.8%) patients with enlargements of the central canal of the spinal cord, 2 (10.5%) patients with cord atrophy, 3 (15.8%) patients with cord swelling, and 1 (0.8%) patient with CSF loculation. The lesions showed either eccentric (5, 26.3%) or diffuse (14, 73.7%) distributions (Figure 2). In 5 patients with paradoxical myelitis, none of them were complicated with spinal meningeal enhancement or arachnoiditis, while 4 patients were complicated with an enlargement of the central canal of the spinal cord (Figure 3).

### Predictors of Myelitis

In univariate analysis, the patients with TBM with myelitis more often had a disturbance of consciousness [*p* = 0.002, odds ratio (OR): 10.952, 95% CI: 2.353–50.978] and had a worse disease severity (*p* < 0.001, OR = 14.986, 95% CI: 4.788–46.91). During the treatment, the myelitis group was more likely to have a paradoxical reaction (*p* = 0.008, OR = 4.939,95% CI: 1.525–15.598) (Table 2). In the CSF examinations, the patients with myelitis had a higher CSF protein level (*p* = 0.005,OR = 1.831, 95% CI: 1.199–2.796) (Table 2). In the multivariate analysis, a grade III disease severity on admission (*p* = 0.003, OR = 8.131, 95% CI: 2.080–31.779) and high CSF protein (*p* = 0.033, OR = 1.698, 95% CI: 1.043–2.763) were independent risk factors for myelitis. were independent risk factors for myelitis (Table 2).

**Table 2:** The univariate and multivariate logistic regression analysis for the risk factors of myelitis in patients with TBM.

	**Univarate analysis**		**Multivariable analysis**		
	**OR(95%CI)**	***P*-value**	**β**	**OR (95%CI)**	***P*-values**
**Clinical characteristics**					
Age ≥60	0.424 (0.051–3.497)	0.426	-	-	-
Male	1.501 (0.549–4.109)	0.429	-	-	-
Extracranial tuberculosis	0.427 (0.115–1.579)	0.202	-	-	-
Paradoxical reaction	4.939 (1.525–15.598)	0.008	0.466	1.593 (0.394–6.438)	0.513
Disease severityof grade III	14.986 (4.788–46.91)	<0.001	**2.096**	**8.131 (2.080–31.779)**	**0.003**
Disturbance of consciousness	10.952 (2.353–50.978)	0.002	0.629	1.617 (0.230–11.368)	0.629
Cranial nerve damage	1.057 (0.344–3.342)	0.923	-	-	-
Seizures	2.267 (0.628–8.176)	0.211	-	-	-
Initial brain MRI findings	-	-	-
Infarction	0.094 (0.454–3.880)	0.094	-	-	-
Arterial inflammation	0.837 (0.848–7.998)	0.837	-	-	-
Inflammatory Nodules	0.737 (0.295–4.520)	0.737	-	-	-
Hydrocephalus	0.897 (0.320–5.001)	0.897	1.266	0.320	5.001
Basal exudates	0.605 (0.398–2.886)	0.605	1.067	0.398	2.886
Initial CSF finding			
Pressure	1.005 (0.999–1.112)	0.121	-	-	-
Cell count,	1.001 (0.999–1.003)	0.309	-	-	-
Protein,	1.831 (1.199–2.796)	0.005	**0.529**	**1.698 (1.043–2.763)**	**0.033**
Sugar,	1.019 (0.632–1.643)	0.939	-	-	–
CL mmol/L	0.982 (0.922–1.045)	0.558	-	-	-

*Bold values indicate statistical significance (p < 0.05), Multivariable logistic analysis was used to select the predictors for the variables with a p < 0.05 in univariate analysis*.

*B, regression coefficient; OR, odds ratio; CI, confidence interval; MRI, magnetic Resonance Imaging; CSF, cerebrospinal flfluid; Cl, chloride*.

### Clinical and Neuroimaging Predictors of a Poor Outcome

**Figure 4:**
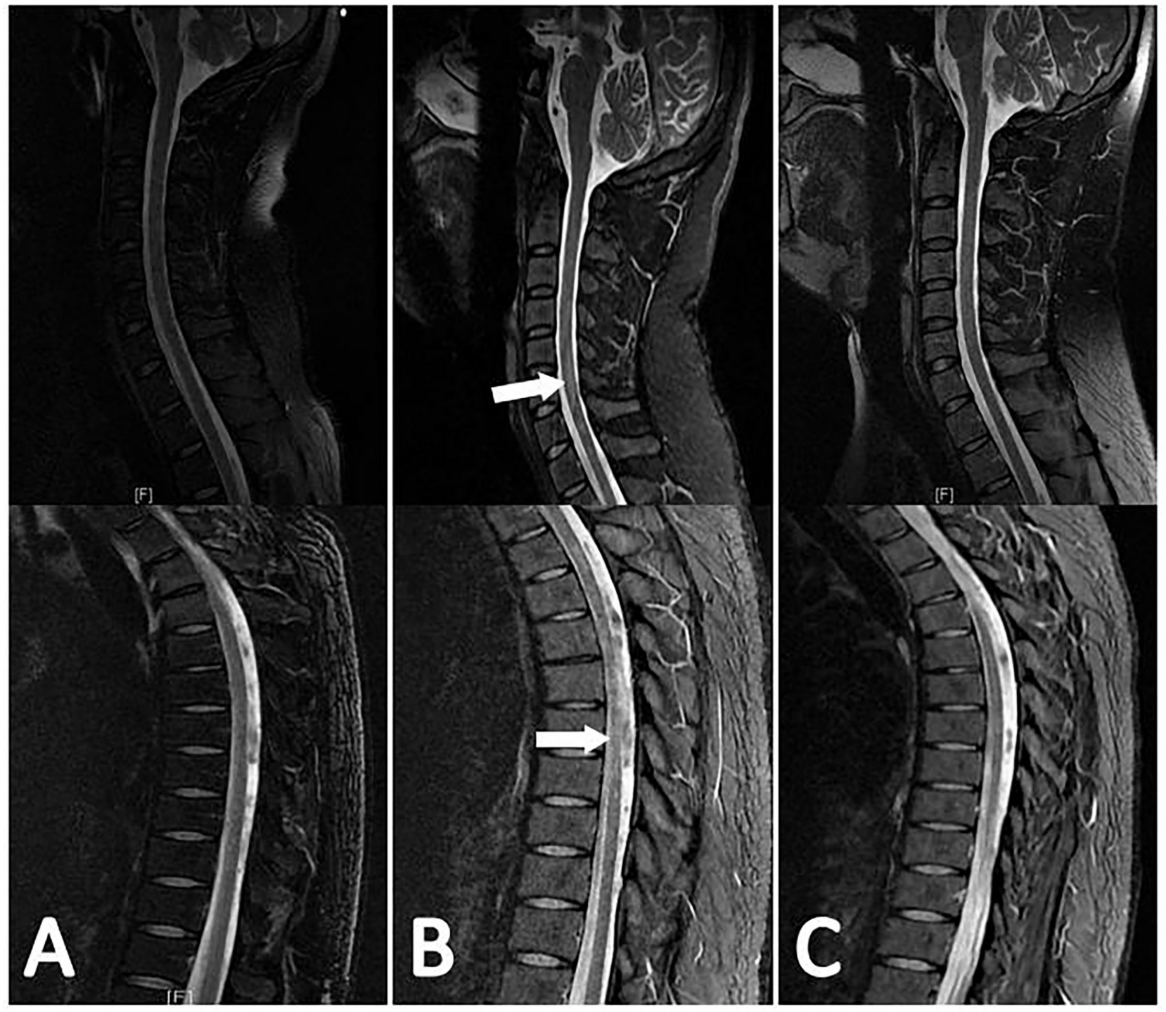
The evolution of myelitis in the same patient is shown in Figure 3. The patient's first spinal MR examination showed no abnormalities **(A)**. 3 months later, there were multiple flaky hyperintensities in the cervical and thoracic spinal cord in the sagittal view of the T2-weighted sequence **(B)**. 4 months later, the original lesion was absorbed **(C)**.

A total of 100 (87.7%) patients had good outcomes and 14 (12.3%) patients had poor outcomes. In the myelitis group, 10 patients (8.8%) had good outcomes, whereas 9 patients (7.9%) had poor outcomes (Table 3). Eight patients had no relief of their spinal cord symptoms and could not walk, 2 patients partially recovered and could walk with some help, and 9 patients completely recovered (Figure 4). In the paradoxical myelitis group, 2 patients completely recovered, 1 partially recovered, and 2 had no relief.

**Table 3:** Clinical and neuromaging characteristic of poor or good outcome.

**Characteristics**	**Good outcome (*n* = 100)**	**Poor outcome (*n* = 14)**	***p-*values**
Paradoxical reaction	46 (46%)	10 (71%)	0.075
Grade III disease severity	16 (16%)	9 (64%)	**<0.001**
Paraparesis	9 (9%)	3 (21%)	0.340
Quadriparesis	7 (7%)	4 (29%)	**0.038**
Pain/paresthesia	5 (5%)	6 (43%)	**<0.001**
Disturbance of consciousness	3 (3%)	5 (36%)	**<0.001**
Bowel or bladder involvement	15(15%)	7(50%)	**0.006**
Cranial nerve damage	26(26%)	3(21%)	0.968
Epilepsy	12 (12%)	2 (14%)	1.000
Brain infarction	25 (25%)	5 (36%)	0.628
Arterial inflammation	15 (15%)	5 (36%)	0.136
Inflammatory Nodules	14 (14%)	2 (14%)	1.000
Hydrocephalus	11 (11%)	4 (29%)	0.173
Basal exudates	42 (42%)	10 (71%)	**0.042**
Myelitis	10 (10%)	9 (64%)	**<0.001**

*Bold values indicate statistical significance (p < 0.05) of χ^2^ test or Fisher's exact test*.

We divided all the patients with TBM into two groups (good outcome and poor outcome) to determine the influencing factors on the prognosis. In the logistic univariate analysis, a grade III disease severity (*p* < 0.001, OR =9.45, 95% CI: 2.798–31.911), quadriparesis (*p* = 0.019, OR = 5.314, 95% CI:1.322–21.355), pain/paresthesia (*p* < 0.001, OR = 14.250, 95% CI: 3.533–57.151), disturbance of consciousness (*p* < 0.001, OR = 17.963,95% CI:3.678–97.729), bowel or bladder involvement (*p* = 0.004, OR = 5.667, 95% CI:1.736–18.492), and myelitis (*p* < 0.001, OR = 16.2, 95% CI: 4.533–57.891) were significantly associated with a poor outcome (Table 4). For the laboratory parameters, no differences were found in the CSF tests, such as the cell counts, protein level, glucose, and chlorine, or the serum laboratory tests, including CRP, ESR, blood glucose, blood sodium, and chlorine (data not shown).

**Table 4:** The univariate and multivariate logistic regression analysis for the risk factors of poor outcome in patients with TBM.

	**Univarate analysis**		**Multivariable analysis**		
	**OR (95%CI)**	***P*-value**	**β**	**OR (95%CI)**	***P*-value**
Paradoxical reaction	2.935 (0.8634–9.985)	0.085	-	-	-
Grade III disease severity	9.450 (2.798–31.911)	<0.001	0.230	1.258 (0.168–9.425)	0.823
Paraparesis	2.758 (0.648–11.741)	0.170	-	-	-
Quadriparesis	5.314 (1.322–21.355)	0.019	−1.412	0.244 (0.026–2.332)	0.220
Pain/paresthesia	14.250 (3.533–57.151)	<0.001	1.084	2.957 (0.346–25.258)	0.322
Disturbance of consciousness	17.963 (3.678–97.729	<0.001	**2.536**	**12.625 (1.092–145.903)**	**0.042**
Bowel or bladder involvement	5.667 (1.736–18.492)	0.004	−0.391	0.677 (0.091–5.020)	0.702
Cranial nerve damage	0.776 (0.201–0.302)	0.714	-	-	-
Epilepsy	1.222 (0.243–6.138)	0.807			
Brain infarction	1.622 (0.497–5.299)	0.423	-	-	-
Arterial inflammation	3.074 (0.904–10.451)	0.072	-	-	-
Inflammatory Nodules	1.000 (0.202–4.955)	1.000	-	-	-
Hydrocephalus	3.164 (0.847–11.823)	0.087	-	-	-
Basal exudates	3.393 (1.001–11.563)	0.050	-	-	-
Myelitis	16.2 (4.533–57.891)	<0.001	**2.588**	**13.297 (1.283–137.812)**	**0.030**

*Bold values indicate statistical significance (p < 0.05), Multivariable logistic analysis was used to select the predictors for the variables with a p < 0.05 in univariate analysis*.

*B, regression coefficient; OR, odds ratio; CI, confidence interval*.

The multivariate regression analysis showed that myelitis (*p* = 0.030, OR = 13.237, 95% CI: 1.283–137.812) and disturbance of consciousness (*p* = 0.042, OR = 12.625, 95% CI: 1.092–145.903) were independent risk factors for poor outcomes (Table 4).

## Discussion

We observed that 16.7% of the patients with TBM either initially had myelitis or paradoxically developed myelitis. Disturbance of consciousness, the severity of disease, a paradoxical reaction, and the CSF protein level were related to myelitis. The degree of disease severity on admission was an independent risk factor for the occurrence of myelitis. A higher degree of disease severity and high CSF protein were also related to paradoxical myelitis. Our findings further found that myelitis independently contributed to the disability of the patients with TBM during the follow-up, which, possibly, was often overlooked or covered up by brain symptoms.

We found that 16.7% of the patients with TBM developed myelitis, as identified by spinal MR. This incidence is higher than that in most previous reports. A New Zealand study found that the incidence of overall spinal cord lesions was only 3% in 104 patients with TBM (25). A systematic review summarizing the spinal cord complications in cases before 2015 found that myelitis occurred in only 8% of 147 patients with TBM (15). In India, the incidence of myelitis (22.5%) in patients with TBM was comparable to our results (16). The possible reason may be due to the longer mean duration of illness before the initiation of efficient treatment and the inclusion of more severe patients; that is, patients with stage III disease were included in this study.

The pathogenesis of myelitis in patients with TBM can be divided into two categories: direct infection of MTB and immune response. First, MTB spreads in the blood and directly invades the spinal cord parenchyma or meninges, causing an intramedullary tuberculoma or vascular inflammation (26). We found that nearly one-third of patients with myelitis complicated with an intramedullary tuberculoma had evidence of direct invasion of MTB into the spinal cord. Our results also showed that more than three spinal segments were involved in patients with TBM with myelitis. The involved segments were usually the cervical and thoracic sections of the spinal cords, which was obviously inconsistent with the vascular distribution of the spinal cord. The activation of the immune response might play an important role in the pathogenesis of myelitis. The study by Hughes and his colleagues suggested that MTB had similar antigens to myelin basic protein (27). There was obvious inflammation and demyelination in sensitized guinea pigs after BCG injection. In addition, lymphocytes sensitized by MTB can recognize and attack the myelin sheath, which suggests that a delayed hypersensitivity induced by MTB may be one of the reasons for the demyelination (28, 29). This evidence emphasized that the immune response may expand the damage caused by a focal infection of MTB.

In this study, ~20% (5/19) of the patients with myelitis developed the myelitis paradoxically. The spinal MRIs showed that the lesions were involved with more than 3 segments of the cervical and thoracic spinal cord, and the MRIs were usually without spinal enhancement and arachnoiditis. This result suggested that compared with direct invasion, an overactivated immune response may occupy a more dominant position in the development of paradoxical myelitis. It is worth mentioning that 80% (4/5) of the patients have paradoxical myelitis complicated with an enlargement of the central canal of the spinal cord, presumably because the focal scarring caused by perivascular inflammation may block the circulation of the CSF, thus forcing CSF into the central canal of the spinal cord via the Virchow–Robin spaces (30). On the other hand, longitudinal spinal inflammatory lesions and an enlarged spinal cord central canal are both typical imaging manifestations of neuromyelitis optica (NMO). In our previous studies, two patients with TBM developed NMO-like symptoms, including myelitis in 1 patient and optic neuritis in the other patient (31). Because aquaporin 4 antibodies and myelin oligodendrocyte glycoprotein antibodies were not tested in our patients, an NMO-like autoimmunity could not be completely excluded. There might be an immune mechanism that is similar to NMO in paradoxical myelitis. A paradoxical reaction is considered to be an excessive immune response. When anti-TB drugs are effective, a large number of TB antigens are released, inducing an excessive inflammatory response against TB antigens and then causing inflammation (6). The occurrence of myelitis should be differentiated from the progression of the disease and a treatment failure. Timely identification and early treatment are important to reduce the occurrence of complications.

Patients with grade III disease severity on admission and a high CSF protein level were more likely to have myelitis. Myelitis itself, leading to paralysis, contributes to the disease severity and could be an indicator of severe disease. More interestingly, not only myelitis that initially developed, but also paradoxical myelitis is both related to the disease severity. A previous study found that a tenfold higher MTB load was associated with an increased disease severity and increased CSF neutrophil and cytokine concentrations (32). Another study that evaluated 67 patients with TBM (94.4% with grade II or III) found that these severe patients with TBM showed a strong blood myeloid response (33). In patients with a higher disease severity, after the initiation of anti-TB treatment, more TB antigens could be released, and excessive inflammation could be induced, leading to the development of paradoxical myelitis.

Garg et al. analyzed 1,078 reports (including case reports) on TBM from 1947 to 2015. The results showed that spinal cord atrophy, vacuoles, multiple complications, and syringomyelia were often associated with a poor prognosis (15). However, the prognosis of myelitis has not been systematically reviewed. In this study, we found that myelitis was a strong predictor of a poor prognosis in patients with TBM. A timely identification and early treatment are important. In particular, we need to be alert to any new signs of spinal cord injury after the initial treatment starts and conduct spinal MRI examinations in a timely manner.

This study has some limitations. Due to the inherent bias of retrospective studies and single-center studies, the extrapolation of the results to a larger population will still be limited. In this study, spinal cord MRIs were only performed in patients with spinal cord injury symptoms and not in all patients with TBM, which may omit some cases of myelitis in which spinal cord injury symptoms were masked. We excluded some patients with symptoms of spinal cord injury but without spinal cord MR examination for various reasons, which may have an impact on the final results, especially on the incidence of myelitis.

## Conclusion

In summary, myelitis is a common complication of TBM. It is caused not only by the progression of the disease before treatment but also by an abnormal immune response during treatment. Grade III disease severity on admission and high CSF protein on admission are related to the development of myelitis. Patients with myelitis often have poor outcomes. Further research on the pathogenesis of TB myelitis will be helpful to provide potential effective therapy to these patients.

## Data Availability Statement

The raw data supporting the conclusions of this article will be made available by the authors, without undue reservation.

## Ethics Statement

The studies involving human participants were reviewed and approved by the Ethics Committee of the Third Affiliated Hospital of Sun Yat-sen University. The ethics committee waived the requirement of written informed consent for participation.

## Author Contributions

TL, LS, and YiJ designed the study. YuJ, XX, and JL collected and analyzed the clinical data. ZG and YL reviewed the MRI data and prepared the figures. YuJ drafted the manuscript. BL reviewed the manuscript. TL and BL approved the final version of the manuscript. All authors contributed to the article and approved the submitted version.

## Conflict of Interest

The authors declare that the research was conducted in the absence of any commercial or financial relationships that could be construed as a potential conflict of interest.

## Publisher's Note

All claims expressed in this article are solely those of the authors and do not necessarily represent those of their affiliated organizations, or those of the publisher, the editors and the reviewers. Any product that may be evaluated in this article, or claim that may be made by its manufacturer, is not guaranteed or endorsed by the publisher.
